# Ammonia oxidizing archaea and bacteria respond to different manure application rates during organic vegetable cultivation in Northwest China

**DOI:** 10.1038/s41598-023-35134-3

**Published:** 2023-05-18

**Authors:** Zhan Wang, Yinkun Li, Wengang Zheng, Yuru Ji, Minjie Duan, Li Ma

**Affiliations:** 1 Research Centre of Intelligent Equipment, Beijing Academy of Agriculture and Forestry Sciences, Beijing, 100097 China; 2 Guyuan Branch, Ningxia Academy of Agricultural and Forestry Sciences, Guyuan, 756000 China; 3 Beijing Key Laboratory of Ecological Function Assessment and Regulation Technology of Green Space, Beijing Urban Ecosystem Positioning Observation and Research Station, Beijing Institute of Landscape Architecture, Beijing, 100102 China; 4 Wuzhong National Agricultural Science and Technology Park Management Committee, Wuzhong, 751100 Ningxia China

**Keywords:** Element cycles, Microbial ecology, Soil microbiology

## Abstract

Ammonia oxidization is a critical process in nitrogen cycling that involves ammonia oxidizing archaea (AOA) and bacteria (AOB). However, the effects of different manure amounts on ammonia-oxidizing microorganisms (AOMs) over the course of organic vegetables production remains unclear. We used the *amoA* gene to evaluated AOMs abundance and community structure in organic vegetable fields. Quantitative PCR revealed that AOB were more abundant than AOA. Among them, the *amoA* copy number of AOB treated with 900 kgN ha^−1^ was 21.3 times that of AOA. The potential nitrification rate was significantly correlated with AOB abundance (*P* < 0.0001) but not with AOA, suggesting that AOB might contribute more to nitrification than AOA. AOB sequences were classified into *Nitrosomonas* and *Nitrosospira,* and AOA into *Nitrosopumilus* and *Nitrososphaera*. *Nitrosomonas* and *Nitrosopumilus* were predominant in treatments that received manure nitrogen at ≥ 900 kg ha^−1^ (52.7–56.5%) and when manure was added (72.7–99.8%), respectively, whereas *Nitrosospira* and *Nitrososphaera* occupied more than a half percentage in those that received ≤ 600 kg ha^−1^ (58.4–84.9%) and no manure (59.6%). A similar manure rate resulted in more identical AOMs’ community structures than greater difference manure rate. The bacterial *amoA* gene abundances and ratios of AOB and AOA showed significantly positive correlations with soil electrical conductivity, total carbon and nitrogen, nitrate, phosphorus, potassium, and organic carbon, indicating that these were potential key factors influencing AOMs. This study explored the AOMs’ variation in organic vegetable fields in Northwest China and provided a theoretical basis and reference for the subsequent formulation of proper manure management.

## Introduction

Organic cultivation is one of the leading agricultural patterns worldwide, including vital organic vegetable cultivation^1,2^. The global organic agricultural cultivation area continuously increases and reached over 74.0 million hectares in 2020^3^. Since 2015, Ningxia has served as a major organic vegetable cultivation province with an inland area of 6670 ha for organic vegetable export^4,5^. The organic vegetable cultivation area has been increasing in Ningxia due to unique climatic conditions such as extended daylight, considerable temperature differences, intense solar radiation, and favorable primary conditions, including advanced animal husbandry, rich manure resources, and no pollution^6,7^. A large amount of organic fertilizer is usually applied during organic vegetable production to enrich the soil. However, nitrogen is often still deficient during crop growth because the nitrogen in organic fertilizer is mainly organic nitrogen, which needs to be decomposed into inorganic nitrogen before it can be absorbed and utilized by crops^4,5^.

Manure produced by animal husbandry is the primary fertilizer nitrogen source for organic vegetable production. Several organic nitrogenous compounds in manure are easily transformed into ammonia through ammonification, which can then be oxidized to nitrate via nitrification^8^. Ammonia oxidation is the first and rate-limiting nitrification step because of its final product, nitrate^9^. The main reasons for excessive manure application during vegetable production are the lack of knowledge and control of the amount of nitrogen supplied by manure and changes in the critical microbial community structure^4,7^. The ammonia oxidation process was long thought to be primarily restricted by ammonia-oxidizing bacteria (AOB)^10^. Known AOB are categorized into two monophyletic groups, *Gammaproteobacteria*, comprising members of the genus *Nitrosococcus*, and *Betaproteobacteria*, including the genera *Nitrosospira* and *Nitrosomonas*^11,12^. However, this long-held view changed after the gene encoding ammonia monooxygenase (*amoA*) was discovered in ammonia-oxidizing archaea (AOA) within the phylum *Thaumarchaeota*^13^. *Thaumarchaeota* was shown to aerobically oxidize ammonia to nitrite, indicating that both AOA and AOB can be responsible for the conversion of ammonia^14^. Since then, the abundance, diversity, and community structures of ammonia-oxidizing microorganisms (AOMs) in distinct environments have been extensively researched, indicating they often coexist in various habitats^12,15^.

The AOA and AOB community structures are essential for assessing nitrification. The AOA and AOB distribution reportedly depends on habitat type^16,17^. AOA outnumber AOB in many ecosystems, such as drinking water bioreactors, river sediment, river water, acidic soils, terrestrial environments, and paddy soils^18–20^. Additionally, AOB, rather than AOA, may dominate nitrogen-rich grassland soils, agricultural soils, lakes, and rivers^21,22^. These differences may be due to the effect of environmental factors and the distinct habitat types.

Moreover, AOA’s and AOB’s abundance and diversity are reportedly affected by many abiotic factors, including pH, ammonium levels, electrical conductivity, and organic matter content^20,23,24^. Generally, ammonia, in ammonia oxidation substrate, has been considered the primary factor in manipulating the distribution of AOMs^25^. AOB growth was favored at high ammonium levels in field soils. Moreover, freshwater river studies reported that the AOMs' community distribution was related to organic matter and ammonia. pH is another essential factor influencing the AOM's distribution^26,27^. AOB reportedly become more competitive in environments with higher ammonium concentrations, while AOA may dominate ammonia oxidation in ecosystems with low pH and ammonium concentrations. In low nitrogen and strong acidic environments, AOA was the main nitrification driver, while AOB primarily have functional activity in high nitrogen, neutral, and alkaline environments^28,29^. The varied AOA and AOB communities’ responses to different niches and physiochemical characteristics highlight the need for an increased focus on agricultural research to improve manure application for organic vegetable production systems.

Therefore, this study designed a field plot experiment in which soils were amended with different amounts of manure. The objectives of this study were to compare the relative contributions of AOA and AOB to the process of ammonia oxidation and the changes in AOM abundance and community structure in response to different amounts of manure and to determine which major soil physicochemical variables affect AOA and AOB segregation in the organic vegetable field.

## Material and methods

### Site description and soil sampling

This study was conducted at the Wuzhong National Agricultural Science and Technology experimental station, located in Ningxia province, China (106°6′26″ E, 37°57′10″ N). The station is situated in the semi-humid and semi-arid temperate continental monsoon climate with an average annual temperature of 5.3–9.9 °C and a yearly average of 105–163 frost-free days. According to the international classification system World Reference Base, the soil in the area is classified as sandy soil^30^. The field capacity and soil bulk densities were 29.7 m^3^ m^−3^ and 1.65 g cm^−3^, respectively. The total nitrogen organic matter, available phosphorous, and potassium in the 0–20 cm soil layer were 0.20, 1.57, 0.013, and 0.13 g kg^−1^, respectively.

The experiment was carried out from May to October 2016 in three growth cycles for Chinese Flowering Cabbage (*Brassica parachinensis L. ssp. Chinensis var. utilis*; Fig. S4). Following local production methods, the first, second, and third round crops were sowed on May 13, July 15, and September 10, respectively, and harvested on June 23, Augustus 18, and October 22, respectively^4,6,7^. The experiment employed a randomized complete block design with five manure dosage treatments, replicated thrice. Each treatment was applied to a plot with an area of 10 × 4 m with a buffer zone of 0.5 m surrounding each plot to explore the effect of manure dosage on AMOs^7,31^. The treatment dosages were as follows: 300 kgN ha^−1^ (M1), 600 kgN ha^−1^ (M2), 900 kgN ha^−1^ (M3), 1200 kgN ha^−1^ (M4), and 0 kgN ha^−1^ (M0, the control)^4,31^. Cow dung was used as an only-nitrogen fertilizer source and was applied once a year before the first crop was planted according to the local customary production method^6^. The cow dung has a pH of 6.9 and contained 12.9 g kg^−1^ total nitrogen, 10.1 g kg^−1^ available potassium, 1.2 g kg^−1^ available phosphorous, and 195.0 g kg^−1^ organic matter.

Topsoil samples (0–20 cm) were collected from each plot in October 2016 after harvesting the Chinese Flowering Cabbage. A total of six soil cores (5 cm) were taken from each field, and samples of the same manure dosage area were mixed thoroughly to form one composite sample. After collection, all samples were divided into three parts; one was transported to the laboratory on dry ice and stored at − 80 °C for molecular analysis, and the second was kept at 4 °C for 24 h until the potential nitrification determination was completed. The third was used to determine the soil's physicochemical properties.

### Potential nitrification rate measurement

As previously described^32^, 300 µM of 2-phenyl-4, 4, 5, 5,-tetramethylimidazoline-1-oxyl 3-oxide (PTIO) was used in the current study since a 200 µM PTIO concentration may be insufficient in inhibiting AOA while a concentration of 400 µM might result in inhibiting AOB. A total of 4 µM 1-octyne was added to inhibit AOB^33^. The PNR was measured as previously described^34,35^. Briefly, each sample was divided into three subsamples. All subsamples (10.0 g fresh soil) were placed in 120-mL serum vials with phenolic caps and butyl stoppers containing 20 mL deionized water and 1 mM NH_4_Cl. The samples were shaken at 24 °C (110 rpm) for 2 h in the dark to prepare soil slurries. This soil slurry was then exposed to 4 µM 1-octyne, 300 µM PTIO, or none. Then, 15 mg mL^−1^ KClO_3_ was added to all samples to inhibit NO_2_^−^ oxidation. Soil slurry (1 mL) samples were collected for NO_2_^−^ analysis at 2, 12, 24, 48, and 72 h after adding 1-octyne, PTIO, and KClO_3_. The aliquots were centrifuged at 15,000 × g for 5 min. The supernatant were frozen until NO_2_^−^ measurement under unifying conditions. However, no significant NO_3_^−^ accumulation was observed after KClO_3_ was added to inhibit NO_2_^−^ oxidation in this study, indicating that NO_2_^−^ oxidation was satisfactorily inhibited.

The PNR was calculated from the rate of the linear accumulation in NO_2_^−^ concentrations over time. The PNR of AOA and AOB was calculated by subtracting the NO_2_^−^ accumulation in the PTIO or 1-octyne treatment from the values measured with only KClO_3_. The NO_3_^−^ and NO_2_^−^ concentrations were measured using a flow injection autoanalyzer (SKALAR, San++, Netherlands).

### DNA extraction

Soil DNA was extracted using an EZNA DNA Soil kit (Omega Bio-Tek Inc., Norcross, GA, USA) per the manufacturer’s protocols. The extracted DNA was visualized using 1% agarose gel during electrophoresis, diluted in TE buffer (10 mM Tris–HCl, 1 mM EDTA, pH 7.0), and stored at − 20 °C before use.

### Quantitative PCR

The *amoA* gene’s abundance in AOA and AOB was quantified by real-time PCR using the Arch-amoAF/Arch-amoAR primers for the AOA *amoA* genes and amoA1F/amoA2R for the AOB *amoA* genes. All qPCR assays were performed in triplicate with an ABI PRISM R 7500 Sequence Detection System (Applied Biosystems, Waltham, MA, USA) using the SYBR Green I method. The 20 mL qPCR reaction system contained 10 mL FastStart Universal SYBR Green Master (ROX) (Roche, NJ, USA), 0.4 µL 10 µM each forward and reverse primer, 7.2 µL sterilized MilliQ water, and 2 µL standard or extracted soil DNA. Amplification was conducted in a LightCycler® 480 (Roche Applied Science, Penzberg, Germany) as follows: 95 °C for 10 min, followed by 40 cycles of 15 s at 95 °C, 45 s at 54 °C and 60 s at 72 °C for AOA, or 40 cycles of 15 s at 95 °C and 2 min at 58 °C for AOB. Standard melting curves were generated using 10-fold serial dilutions of a plasmid containing the AOA or AOB target gene inserts.

### Barcoded pyrosequencing

Pyrosequencing of the AOA and AOB *amoA* gene was performed using the specific primer pairs Arch-amoAF/Arch-amoAR and amoA1F/amoA2R, respectively. Barcodes were ligated to the 5′-ends of the primers to distinguish each sample. Each sample was then PCR amplified in 25 µL reactions containing 10 ng DNA under the following conditions: 98 °C for 5 min for initial denaturation, followed by 30 cycles of 98 °C for 30 s, 50 °C for 45 s, and 72 °C for 60 s, and final extension at 72 °C for 7 min. After purification with an Agarose Gel DNA purification kit (TaKaRa, Dalian, China), pyrosequencing was conducted using a 454 Life Science Genome Sequencer FLX Titanium instrument (Roche, NJ, USA) by Personal Biotechnology Co., Ltd., Beijing, China.

### Statistical analysis

The raw sequences obtained by pyrosequencing that matched the barcodes and primers were retained. Data were processed using QIIME Pipeline Version 1.9.0 (http://qiime.sourceforge.net/)^36^. Briefly, reads with a mean quality score of < 20 or > 200 bp and < 500 bp in length were removed, and the 10 bp barcode was examined to assign sequences to each sample. The sequences were trimmed using the UCHIME algorithm to remove the barcodes and primers^37^. After quality control, USEARCH was performed to cluster the high-quality sequences into OTUs with a 97% similarity cutoff^38^. The richness indices (ACE estimator), diversity indices (Shannon index), and Good's coverage were also processed using QIIME Pipeline Version 1.9.0^36^. Phylogenetic analysis was performed using MEGA6^39^, while redundancy analysis was performed using CANOCO for Windows (version 4.5) to identify the correlations between soil parameters and microbial communities^40^. Pearson's correlation analysis of the abundance, transcript abundance, PNR, and environmental factors was performed using the SPSS statistics software (version 19.0 for Windows). Principal coordinate analysis (PCoA) and analysis of similarities (ANOSIM) were used to measure community similarity based on the algorithm of the Bray–Curtis matrix, and 999 Monte Carlo permutations were used to assess the statistical significance of diversity metrics.

### DNA sequence accession numbers

The raw data generated from pyrosequencing were submitted to the National Center for Biotechnology Information Sequence Read Archive database under accession numbers SRP425288 and SRP425614.

## Results

### AOA and AOB abundance and potential nitrification rates

In organic vegetable fields, the abundance of soil AOA and AOB *amoA* genes were 6.63 ± 0.24 × 10^5^–11.14 ± 0.90 × 10^5^ and 1.20 ± 0.11 × 10^6^–18.99 ± 0.99 × 10^6^ copies/g of dry soil, respectively (Table 1). The AOA *amoA* abundance initially decreased, then increased with increasing manure levels, whereas the *amoA* abundance of AOB increased continuously. In addition, the ratios of AOB and AOA *amoA* gene copy numbers varied from 1.08 to 20.67 among all treatments and decreased in the order of M4 > M3 > M2 > M1 > M0, indicating that the *amoA* copy numbers of AOB were consistently greater than those of AOA (Table 1).

**Table 1:** Basic properties of the sandy loam soil under different fertilization treatments.

Sample	EC (cm^−1^)	pH	TC (g kg^−1^)	TN (mg kg^−1^)	NH_4_^+^ (mg kg^−1^)	NO_3_^−^ (mg kg^−1^)	AP (mg kg^−1^)	AK (mg kg^−1^)	TOC (g kg^−1^)	AOA (copy number 10^5^ g^−1^ soil)	AOB (copy number 10^6^ g^−1^ soil)	AOB/AOA abundance	AOA PNR (mg NO_2_^−^–N kg^−1^ soil h^−1^)	AOB PNR (mg NO_2_^−^–N kg^−1^ soil h^−1^)	Total PNR (mg NO_2_^−^–N kg^−1^ soil h^−1^)
M0	179.6	8.07	11.1	333.0	28.52	1.54	29.52	177.18	2.85	11.14	1.20	1.08	0.22	0.79	1.01
M1	187.8	8.16	12.3	433.0	28.75	3.39	60.89	194.58	4.27	7.35	4.58	6.23	0.52	1.56	2.08
M2	213.4	8.12	12.9	461.0	28.93	4.52	98.71	218.26	5.42	6.63	8.21	12.38	1.03	2.20	3.23
M3	227.3	8.21	13.6	530.0	28.78	6.80	144.21	235.51	7.35	7.85	16.70	21.28	1.50	2.71	4.21
M4	253.0	8.03	15.4	750.0	28.56	7.41	174.32	251.27	9.43	9.19	18.99	20.67	2.46	3.07	5.53

*EC* electrical conductivity, *TC* total carbon, *TN* total nitrogen, *NH*_*4*_^+^ ammonium nitrogen, *NO*_*3*_^*−*^ nitrate nitrogen, *AP* available phosphorus, *AK* available potassium, *TOC* total organic carbon, *M0* without manure application, *M1* annual manure application 300 kgN ha^−1^, *M2* annual manure application 600 kgN ha^−1^, *M3* annual manure application 900 kgN ha^−1^, *M4* annual manure application 1200 kgN ha^−1^, *PNR* potential nitrification rates, *AOA* ammonia-oxidizing archaea, *AOB* ammonia-oxidizing bacteria.

**Figure 1:**
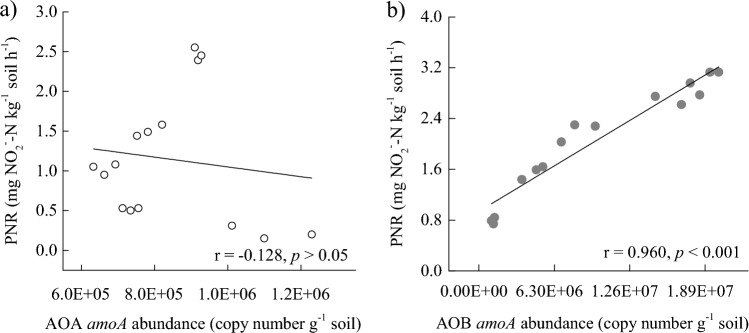
Relationship between AOA and AOB *amoA* gene abundance and potential nitrification rates (PNR) in organic vegetable field soils.

Overall, total potential nitrification rates (PNR) increased as the manure amount increased. The addition of ampicillin to separate the AOA's PNR from the total PNR revealed that the AOB PNR was significantly greater than that of AOA for all samples (*P* < 0.001, Table 1). Pairwise regression analysis revealed that PNR was not correlated with the AOA abundance (Fig. 1a) but was highly correlated with the AOB abundance (*P* < 0.0001, Fig. 1b). These results suggest that AOB might contribute more to nitrification than AOA in organic vegetable fields.

### *amoA* gene diversity

As previously described^23^, the α- and β-diversity of AOA and AOB were measured using *amoA* gene clone libraries. A total of 27,111 high-quality archaeal *amoA* gene sequences were generated, divided into 31 operational taxonomic units (OTUs) using a 3% cutoff (Table 2). Regarding AOB, 84,863 high-quality bacterial *amoA* gene sequences were grouped into 66 OTUs. AOB displayed higher α-diversity (Ace and Shannon indexes) than AOA in all treatments. Individually, the AOA diversity decreased and then increased with increasing manure application, while AOB levels increased continuously. For each sample, AOA had the highest diversity at M1, whereas AOB had the highest variety at M4. The coverage exceeded 0.99 in all treatments, indicating that the *amoA* gene sequences retrieved from the treatments could represent most AOA and AOB communities.

**Table 2:** Diversity of archaeal and bacterial *amoA* gene sequences.

Samples	*amoA* sequence/no. of OTUs		Ace index		Shannon index		Coverage	
	AOA	AOB	AOA	AOB	AOA	AOB	AOA	AOB
M0	27111/24	44378/35	50.30	46.66	1.62	1.78	0.9995	0.9986
M1	15467/22	51618/38	46.14	48.21	1.61	1.81	0.9998	0.9992
M2	13412/17	54822/43	37.00	52.23	1.30	1.92	0.9998	0.9993
M3	16501/17	74171/48	36.72	56.26	1.30	2.05	0.9999	0.9993
M4	18834/14	84863/52	31.73	61.57	1.23	2.23	0.9998	0.9991

*M0* without manure application, *M1* annual manure application 300 kgN ha^−1^, *M2* annual manure application 600 kgN ha^−1^, *M3* annual manure application 900 kgN ha^−1^, *M4* annual manure application 1200 kgN ha^−1^, *AOA* ammonia-oxidizing archaea, *AOB* ammonia-oxidizing bacteria.

**Figure 2:**
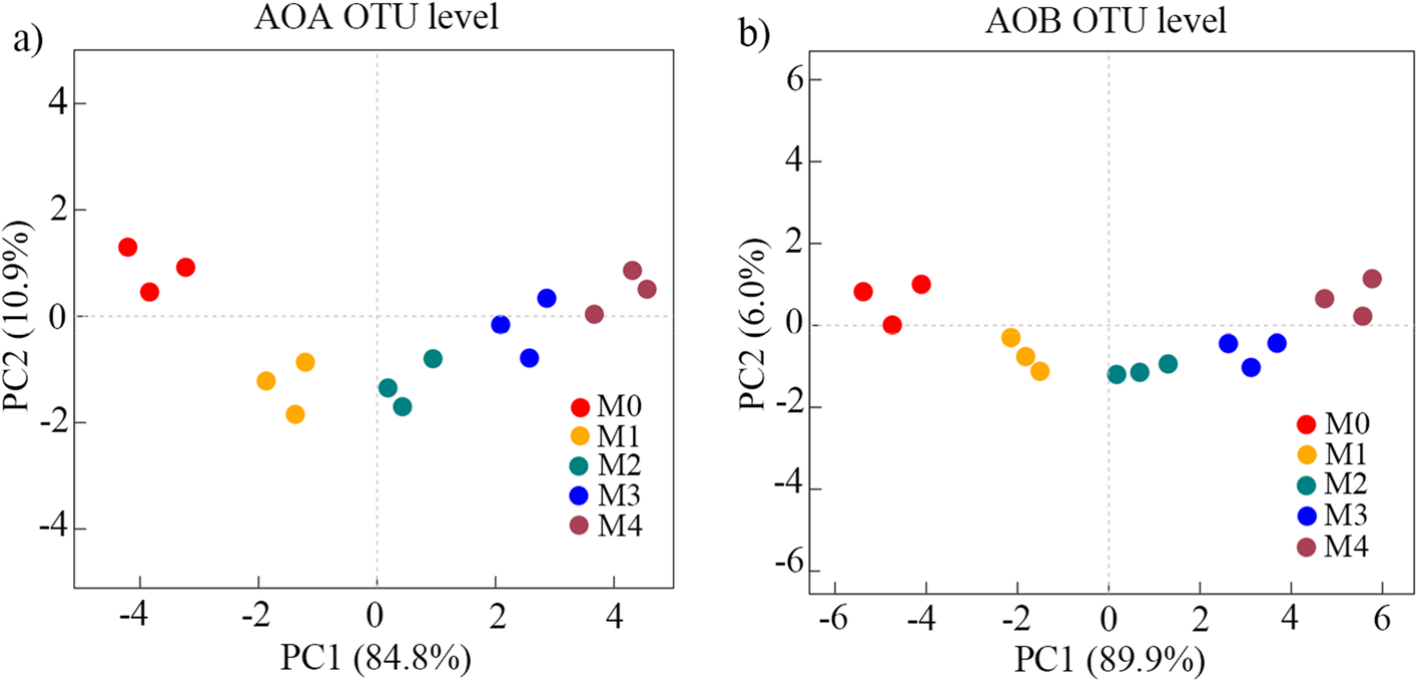
A PCoA plot at OTU level of (**a**) ammonia-oxidizing archaea (AOA); and (**b**) ammonia-oxidizing bacteria (AOB).

Principal coordinate analysis (PCoA) and analysis of similarities (ANOSIM) were used to evaluate the β-diversity of ammonia-oxidizers in organic vegetable fields (Fig. 2). The OTU-based PCoA results indicated that the AOA and AOB communities differed significantly between the fertilization and no fertilization groups (*P* < 0.01). Furthermore, the community structure of AOA and AOB became more similar when the same amount of manure was added and vice versa, indicating that the manure quantity significantly influenced the microbial community structure of organic vegetable fields (*P* < 0.05).

### Phylogenetic analysis and AOA community structure

**Figure 3:**
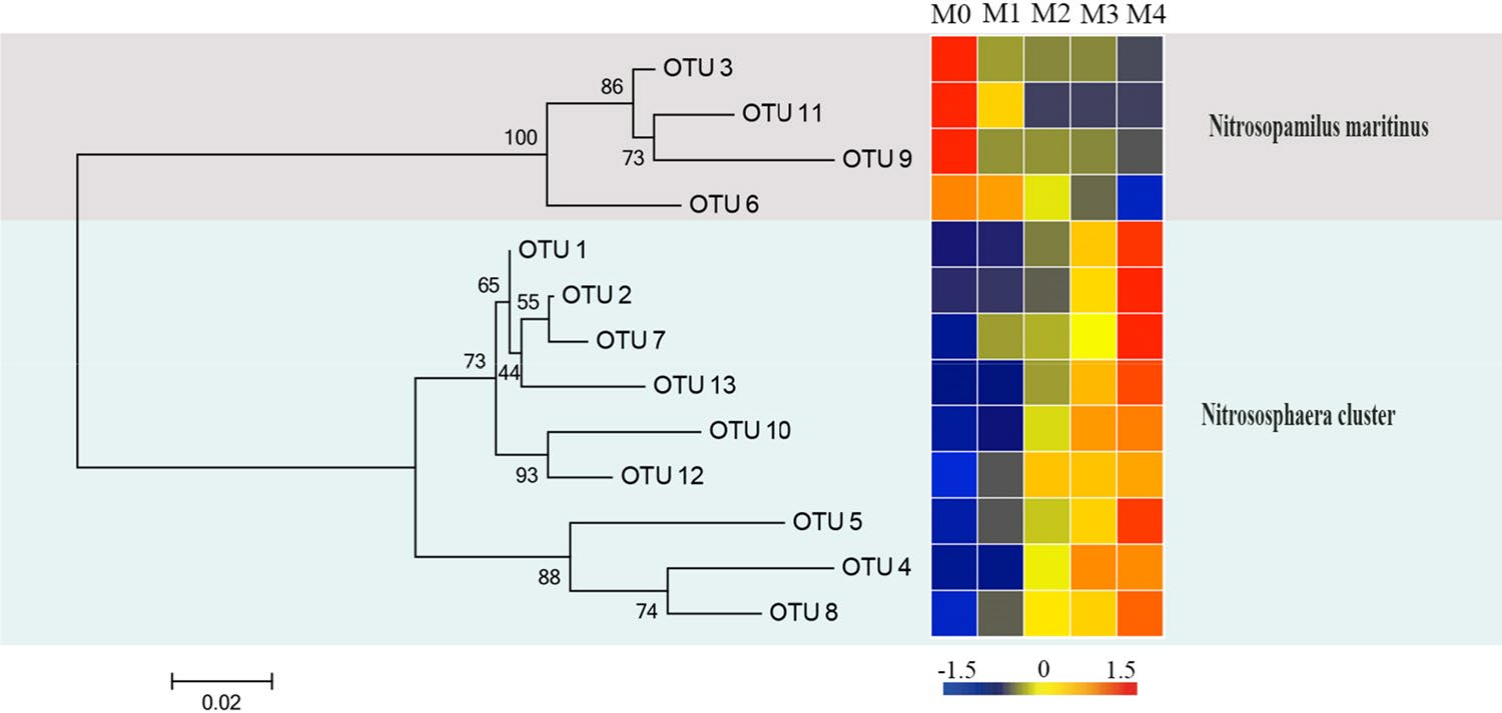
Neighbor-joining phylogenetic tree and community distributions of AOA *amoA* gene sequences from organic vegetable fields under different manure application rates. Bootstrap values > 50% based on 1000 replicates are shown next to each branch, and the scale bar represents a divergence of 0.02 nucleic acid sequences. Each OTU relative proportion value is color-coded in the corresponding heat map legends. M0: without manure application; M1: 300 kgN ha^−1^ annual manure application; M2: 600 kgN ha^−1^ annual manure application; M3: 900 kgN ha^−1^ annual manure application; M4: 1200 kgN ha^−1^ annual manure application.

The AOA phylogenetic tree was constructed using known AOA isolated strains and *amoA* gene sequences of representative OTUs (Figs. 3 and S1). All sequences were grouped into two clusters, namely *Nitrosopumilus* and *Nitrososphaera*. The *Nitrosopumilus* cluster had the greatest abundance group, accounting for 40.4–99.8% of all archaea *amoA* gene sequences with nine OTUs in the organic vegetable field (Fig. S3a). *Nitrososphaera*, which had four OTUs and a relative abundance of 0.2–59.6%, was the second most abundant. Notably, the *Nitrosopumilus* cluster was continuously increasing with the increasing manure application rate, whereas the *Nitrososphaera* cluster was consistently decreasing.

Cluster analysis showed that the AOA community could be classified into two branches (Fig. S3a). The *Nitrosopumilus* cluster was the predominant AOA in manure samples M1, M2, M3, and M4, which accounted for 72.7, 91.9, 94.9, and 99.8% of the total sequences, respectively. For no manure samples, the *Nitrososphaera* cluster occupied more than a half percentage in the M0 treatment. These results imply that AOA communities may be influenced by manure.

### Phylogenetic analysis and AOB community structure

**Figure 4:**
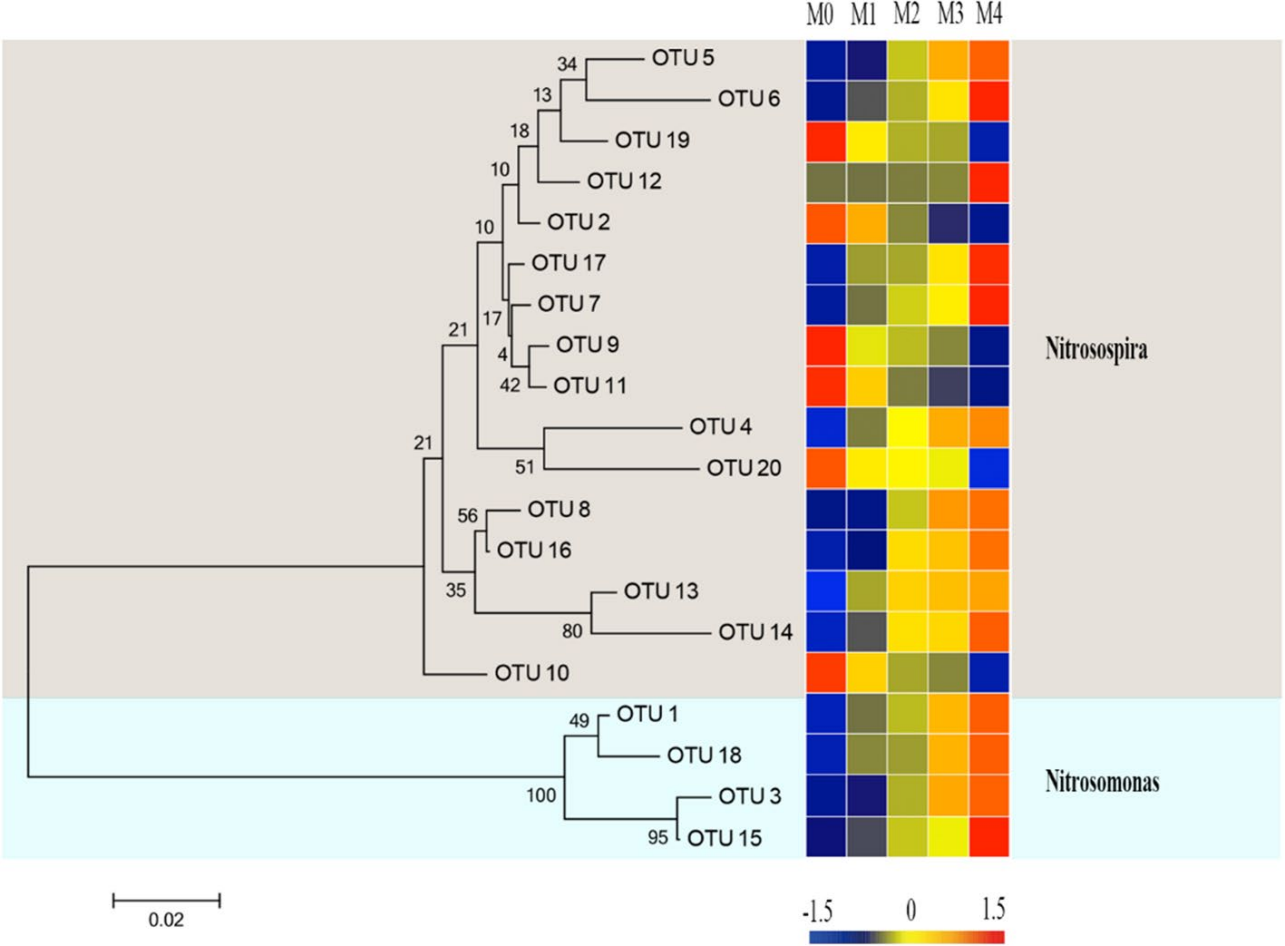
Neighbor-joining phylogenetic tree and community distributions of AOB *amoA* gene sequences from organic vegetable fields under different manure application rates. Bootstrap values > 50% based on 1000 replicates are shown next to each branch, and the scale bar represents a divergence of 0.02 nucleic acid sequences. Each OTU relative proportion value is color-coded in the corresponding heat map legends. M0: without manure application; M1: 300 kgN ha^−1^ annual manure application; M2: 600 kgN ha^−1^ annual manure application; M3: 900 kgN ha^−1^ annual manure application; M4: 1200 kgN ha^−1^ annual manure application.

The AOB *amoA* gene phylogenetic tree showed that all retrieved sequences could be categorized into two major clusters, *Nitrosospira* and *Nitrosomonas* (Figs. 4 and S2). The *Nitrosospira* cluster, containing 16 OTUs and accounting for 43.4–84.9% of all bacterial *amoA* gene sequences, was the most abundant group in the organic vegetable field, followed by the *Nitrosomonas* cluster, which contained four OTUs and had a relative abundance of 15.1–56.6% (Fig. S3b).

Similarity analysis indicated that the AOB community could be clustered into two branches (Fig. S3b). The *Nitrosomonas* cluster accounted for more than half of all bacterial *amoA* gene sequences in the M3 (52.7%) and M4 (56.5%) treatments. Furthermore, the *Nitrosospira* cluster showed relatively higher ratios of 58.4, 67.7, and 84.9% in vegetable field soil samples of M0, M1, and M3, respectively, than the *Nitrosomonas* cluster. These results imply that the manure addition rate had an apparent effect on microbial communities.

### Impact of soil parameters on AOA and AOB communities

**Figure 5:**
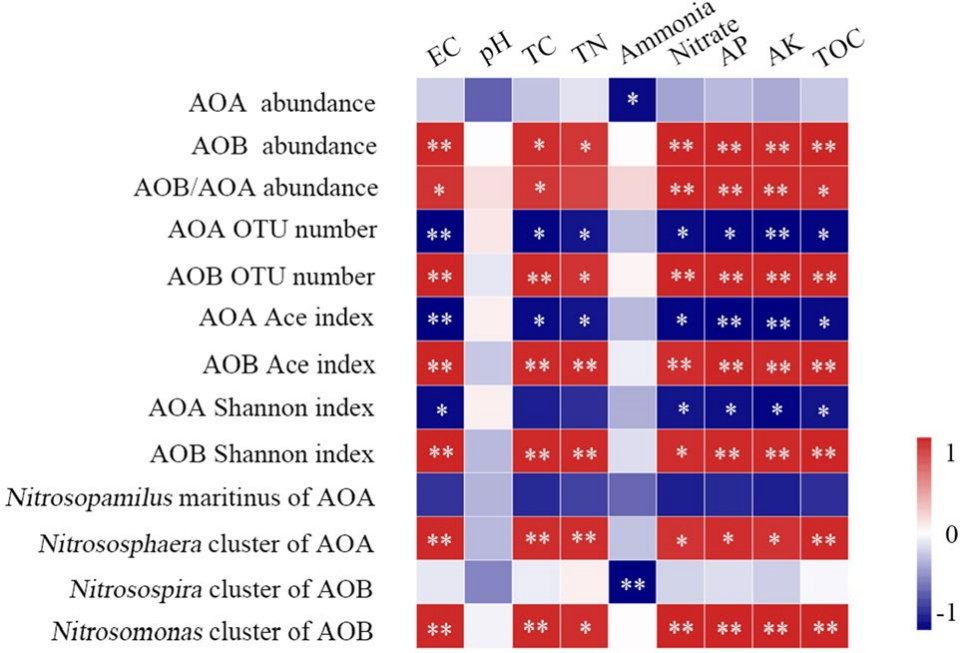
Heat maps visualizing the correlation between soil parameters, ammonia-oxidizing microbial abundance, α-diversity, and predominant taxa. Correlation coefficient values are color-coded in the corresponding heat map legends. **P < 0.01 and *P < 0.05 indicate significant correlation effects at different significance levels. EC: electrical conductivity; TC: total carbon; TN: total nitrogen; AP: available phosphorus; AK: available potassium; TOC: total organic carbon.

Spearman’s correlation analyses were conducted to illustrate the relationship between soil parameters and ammonia-oxidizing microbial abundance, α-diversity, and predominant taxa **(**Fig. 5). The bacterial *amoA* gene abundances in organic vegetable fields showed significant positive correlations with all soil parameters (*P* < 0.05) except for pH and ammonium. In contrast, except for ammonium, no significant correlations were detected between archaeal amoA gene abundances and soil parameters. Additionally, the ratios of AOA and AOB *amoA* gene abundances were positively correlated with electrical conductivity (EC), total carbon (TC), nitrate, available phosphorus (AP), available potassium (AK), and total organic carbon (TOC) (*P* < 0.05). The AOB α-diversity (OTU number, Ace, and Shannon indexes) had notable positive correlations with all soil properties. Interestingly, AOA α-diversity was significantly negatively correlated with soil parameters (*P* < 0.05).

The genera *Nitrososphaera* (AOA) and *Nitrosomonas* (AOB) were also notably and positively correlated with the EC, TC, TN, nitrate, AP, AK, and TOC concentrations (*P* < 0.05). In contrast, the *Nitrosopumilus* (AOA) and *Nitrosospira* (AOB) clusters showed no significant correlations with soil parameters, except ammonium.

## Discussion

Fertilization considerably affects the ammonia-oxidizing microorganism (AOM) distribution in soil^11,12,14^. However, little is known about AOM distribution patterns in organic vegetable fields. This study's quantitative PCR (qPCR) indicated that bacterial *amoA* gene copy numbers were always greater than archaea's under the same manure dosage. These higher ammonia-oxidizing bacteria (AOB) abundances in organic vegetable fields revealed that AOB are more adapted to organic vegetable cultivation conditions and, therefore, might have more versatile metabolisms than ammonia-oxidizing archaea (AOA)^18,41^. Different nitrogen concentrations and pH values reportedly cause differential growth of AOB and AOA, with AOB growing at high nitrogen concentrations in alkaline environments (pH7.0–8.5) and AOA being predominant at low nitrogen concentrations and acidic and neutral conditions (pH3.7–7.0)^21,27,42^. Due to total nitrogen concentration (> 333 mg kg^−1^) and alkalinity of soils (pH > 8.03) were relatively high in this study (Table 1), it is reasonable that higher AOB abundance was detected in the organic vegetable field. With the increase of fertilization within a reasonable range, the crop developed excellently, the root system absorbed more particles and the soil pH increased^43^. For example, the long-term winter wheat and summer corn fertilization experiment on red loam in Hunan Province indicated that after 18 years of continuously adding pig manure (nitrogen dosage 850 kg ha^−1^), the soil pH increased from 5.70 to 6.40^44^. In this study, the amount of 900 kgN ha^−1^ manure used was similar to that added to the Hunan red loam, and the pH increased from 8.07 to 8.21 (Table 1). However, if the manure dosage was too high (such as 1200 kgN ha^−1^ in this study), it was not conducive to crop growth, resulting in soil acidification and pH drop^45^.

Slight changes in pH can impact the AOMs’ community structure^11,12,20^. In this study, soil pH increased with manure dosage (Table 1). This increase may benefit AOB for abundance and diversity but is not conducive to AOA, which may be a fundamental reason for the decreased AOA and the rise of AOB. Furthermore, the AOA and AOB quantities in the organic field had a significantly positive correlation with total carbon **(**TC, *P* < 0.05, Fig. 5). Carbon concentration is also a key factor causing the AOMs abundance to vary. AOMs are reportedly autotrophic microorganisms capable of using carbon dioxide as the only carbon source^46,47^. However, AOMs have been described as mixotrophic or heterotrophic^48,49^. Thus, AOM’s response to carbon sources is very complex, and enhancing carbon can increase the abundance of some members but inhibit that of others^49^. Moreover, two closely-related *Nitrosotalea* strains displayed differences in physiological features, as indicated by variations in growth rate in response to organic compounds^50^. In the organic field soil in the present study, Spearman’s statistical analyses showed that both TC and several AOA and AOB lineages were significantly positively correlated (Fig. 5), supporting this complex response.

The ammonia oxidation rate may or may not be directly related to the AOA and AOB *amoA* abundance. Furthermore, AOB *amoA* abundance is more positively correlated with potential nitrification rates (PNR) than AOA in neutral and alkaline soils^51^. However, AOA communities are more critical than AOB for ammonia oxidization in acidic soils^52^. A paddy soil survey soil revealed that AOA and AOB abundance was significantly positively correlated with PNR, with a stronger correlation for AOB (*r* = 0.724) than AOA (*r* = 0.406)^47^. In the present study, the soil was alkaline (Table 1), and the abundance of AOB was positively correlated with PNR (*r* = 0.960, *P* < 0.001), whereas AOA showed no significant relationships (Fig. 1). This correlation is consistent with the results reported in previous studies, which showed AOB were more suitable for alkaline soils and high nitrogen concentrations than AOA^39,53^. These findings indicate that AOB, rather than AOA, contributed considerably to ammonia oxidization in the organic vegetable field. However, the AOA abundance was lower than that of AOB, and the relationship between relative abundances of different lineages of AOA and AOB and PNR varied greatly (ANOSIM test, data not shown). Therefore, the DNA-SIP method or more detailed experiments (e.g., using different inhibitors) should be used in further studies to assess the contribution of several AOA and AOB lineages to ammonia oxidization in organic vegetable field soil.

All known archaeal genera related to ammonia oxidizers have been classified as members of *Nitrosopumilus, Nitrososphaera*, *Nitrosoarchaeum,* and unclassified as *Thaumarchaeota*^34,54^. Phylogenetic analysis indicated that in the soils of organic vegetable fields, the *Nitrosopumilus* cluster was the main species (72.7–99.8% of all archaeal sequences) for manure-added treatments (M1, M2, M3, and M4). However, in treatments with no added manure, the percentage of *Nitrosopumilus* cluster was much lower, although it was still high (up to 40.4%; Fig. S3a). These findings were consistent with those observed for other soil environments in the Chinese black zone, Greenland, Costa Rica, Namibia, and Austria^55,56^. These findings suggest that *Nitrosopumilus* are the dominant species in organic field soils and most other soils.

Interestingly, the *Nitrosopumilus* cluster continuously increased with the manure rate, whereas the *Nitrososphaera* cluster continuously decreased. Principal coordinate analysis (Fig. 2a) and cluster analysis (Fig. S3a) showed treatments that received similar amounts of manure had more identical AOA community structures. In contrast, greater differences in the manure quantity were associated with more divergent AOA and AOB community structures. These findings imply that manure rate had an evident influence on AOA microbial communities, and *Nitrosopumilus* may be more adaptive to a higher manure quantity, whereas *Nitrososphaera* showed an opposite trend.

The AOA distribution is reportedly closely associated with pH^47,52^. However, we found that the distribution of AOA lineages at the genus level was not significantly related to pH. The inconsistent relationships between soil pH and different AOA lineages may be associated with the various pH ranges tested in previous studies and this study^47^. In this study, the pH value range was narrower (8.03–8.21, Table 1) than in previous research^47,54^, which may lead to the impact of pH on AOA lineage distribution in the organic vegetable soils being masked by other environmental parameters^16,54^. We found that other factors, such as the TC, total nitrogen (TN), electrical conductivity (EC), nitrate, available phosphorus (AP), available potassium (AK), and total organic carbon (TOC) concentrations, were associated with the distribution of specific AOA lineages in organic vegetable soils.

Among bacterial communities, *Nitrosospira* and *Nitrosomonas* were the dominant terrestrial genera^56^. The relative abundance of the *Nitrosospira* genus is greater than that of the *Nitrosomonas* genus in many samples collected from paddy soils^47^, sediment^57^, and water^39^. In contrast, some studies reported that *Nitrosomonas* might be dominant in the black soil zone of northeast China^23^, as well as in aquatic environments and paddy soils^58,59^. These findings suggest that AOB has different distribution characteristics in various settings.

In this study, *Nitrosospira* showed greater abundance in M0, M1, and M2 (58.4–84.9%) samples (organic nitrogen dosage of less than 600 kg ha^−1^), whereas *Nitrosomonas* accounted for more than half of the percentage in the M3 and M4 treatments (organic nitrogen dosage of more than 900 kg ha^−1^). Thus, the AOB community compositions in organic vegetable field soils differed distinctly under different manure application rates. *Nitrosomonas* was more adaptable to large manure amounts, whereas the opposite was true for *Nitrosopira*. However, in organic vegetable fields, the application of different amounts of manure had little effect on soil pH; therefore, the impact of pH on soil microorganisms can be overshadowed by other environmental factors such as large quantities of organic carbon, organic nitrogen, and inorganic nitrogen quantities present in manure. We also observed that the distribution of some AOB lineages at the genus level was positively or negatively related to the EC, TC, TN, nitrate, AP, AK, TOC, and ammonia levels (Fig. 5). Furthermore, AOB were reportedly not significantly correlated with pH but were significantly associated with different environmental factors^23^. This study and previous studies’ results indicate that multiple soil factors influence the distribution of the AOB *amoA* gene in organic vegetable fields.

## Conclusions

The abundance, diversity, and community AOMs structure in organic vegetable field soil receiving different manure amounts were analyzed using the functional marker gene *amoA*. The AOB *amoA* genes showed higher abundance and diversity than the AOA *amoA* genes. There was also a positive relationship between bacterial *amoA* gene copy numbers and PNR, whereas the archaeal *amoA* gene copy numbers were not correlated with PNR. Separation of the AOMs’ total PNR revealed that AOB’s PNR was higher than AOA’s in all samples. Therefore, AOB may dominate the soil nitrifying processes in organic vegetable fields. In addition, AOMs communities analysis in organic vegetable fields revealed they were altered by variations in physicochemical characteristics caused by different manure amounts. The AOA *Nitrosopumilus* and AOB *Nitrosomonas* clusters may be more adaptable to large manure quantities, whereas the opposite was true for the *Nitrososphaera* and *Nitrosopira* clusters. These results provide additional valuable information for future studies investigating nitrogen cycles in organic field systems.

## Supplementary Information

Supplementary Information.

## Data Availability

The data used to support the findings of this study are included within the article.
