# Supplementary material for: Ammonia oxidizing archaea and bacteria respond to different manure application rates during organic vegetable cultivation in Northwest China
Source: Sci Rep. 2023 May 18;13:8064. doi: 10.1038/s41598-023-35134-3 (PMC10195796; doi:10.1038/s41598-023-35134-3)
Supplement: Supplementary file 1 — Supplementary Information. [file 41598_2023_35134_MOESM1_ESM.docx]

**Supplementary information**

Ammonia-oxidizing archaea and bacteria response to different manure application rates during organic vegetable cultivation in Northwest China

Zhan Wang^1,2^, Yinkun Li^1,3,*^, Wengang Zheng^1^, Yuru Ji^1^, Minjie Duan^4^, Li Ma^5^

*^1^ Research Centre of Intelligent Equipment, Beijing Academy of Agriculture and Forestry Sciences, Beijing 100097, China*

*^2^ Guyuan Branch, Ningxia Academy of Agricultural and Forestry Sciences, Guyuan 756000, China*

*^3^ Institute of Environment and Sustainable Development in Agriculture, CAAS, Beijing 100081, China*

*^4^ Beijing Key Laboratory of Ecological Function Assessment and Regulation Technology of Green Space, Beijing Urban Ecosystem Positioning Observation and Research Station, Beijing Institute of Landscape Architecture, Beijing 100102, China*

*^5^ Wuzhong National Agricultural Science and Technology Park Management Committee, Wuzhong, Ningxia, 751100, China*

This file includes:

Number of Figures: 4

* Corresponding Author:

Dr. Yinkun Li E-mail address: [lykun1218@163.com](mailto:lykun1218@163.com).

**
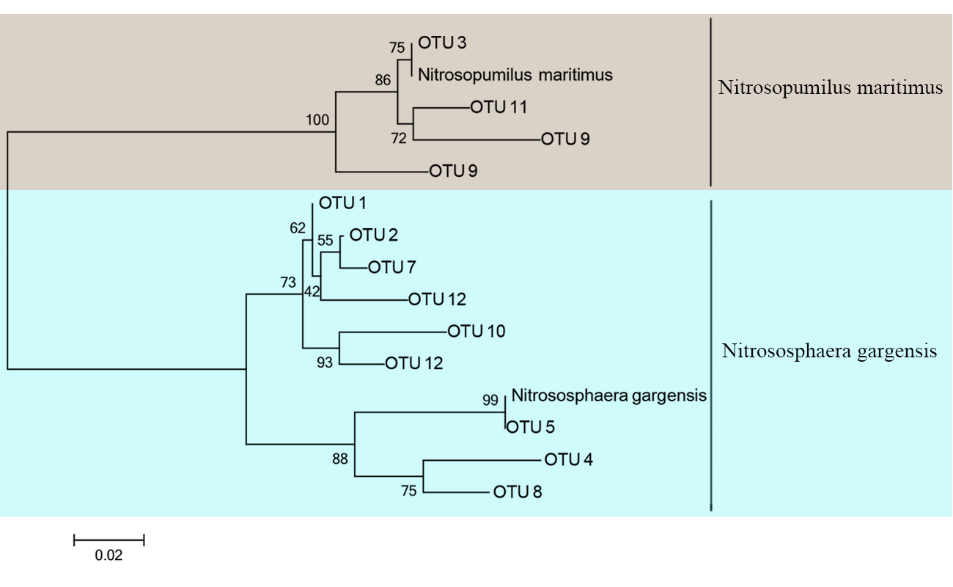
**

**Figure S1.** Neighbor-joining Phylogenetic tree and community distributions of AOA *amoA* gene sequences from organic vegetable field under the different manure application rate. Bootstrap values >50% of 1,000 replicates are shown next to the branch, and the scale bar represents 0.02 nucleic acid sequences divergence. M0: without manure application; M1: annual manure application 300 kgN‧ha^-1^; M2: annual manure application 600 kgN‧ha^-1^; M3: annual manure application 900 kgN‧ha^-1^; M4: annual manure application 1200 kgN‧ha^-1^.


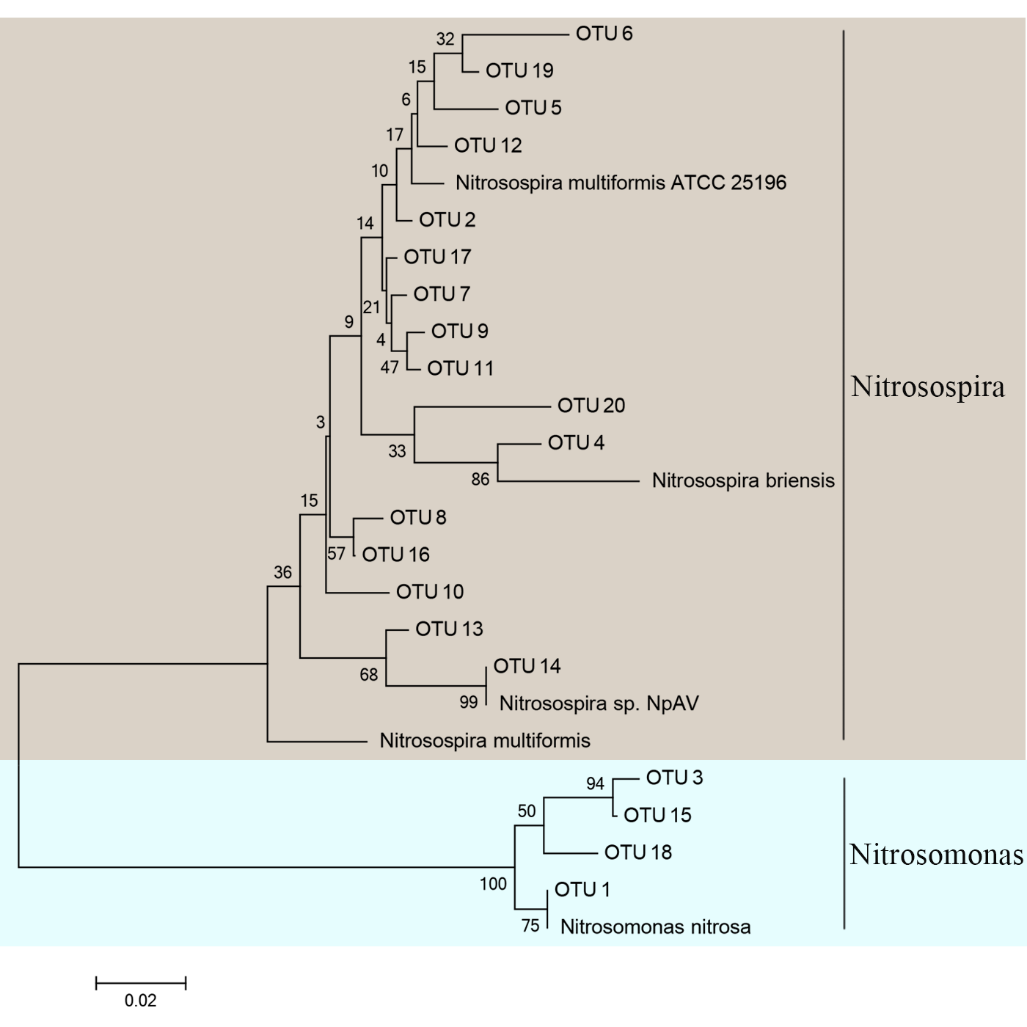


**Figure S2.** Neighbor-joining Phylogenetic tree and community distributions of AOB *amoA* gene sequences from organic vegetable field under the different manure application rate. Bootstrap values > 50% of 1,000 replicates are shown next to the branch, and the scale bar represents 0.02 nucleic acid sequences divergence. M0: without manure application; M1: annual manure application 300 kgN‧ha^-1^; M2: annual manure application 600 kgN‧ha^-1^; M3: annual manure application 900 kgN‧ha^-1^; M4: annual manure application 1200 kgN‧ha^-1^.


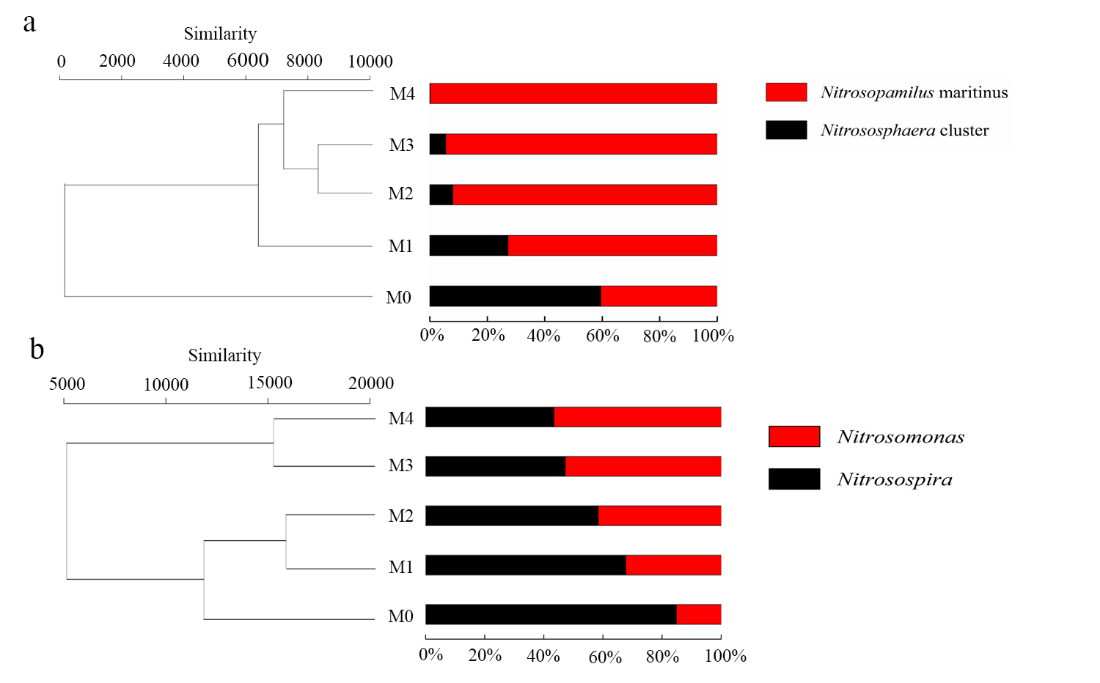


**Figure S3.** Community compositions of AOA (a) and AOB (b) at cluster level in organic vegetable field. Hierarchical clustering of samples is based on the Rray-Curtis similarity algorithm. M0: without manure application; M1: annual manure application 300 kgN‧ha^-1^; M2: annual manure application 600 kgN‧ha^-1^; M3: annual manure application 900 kgN‧ha^-1^; M4: annual manure application 1200 kgN‧ha^-1^.


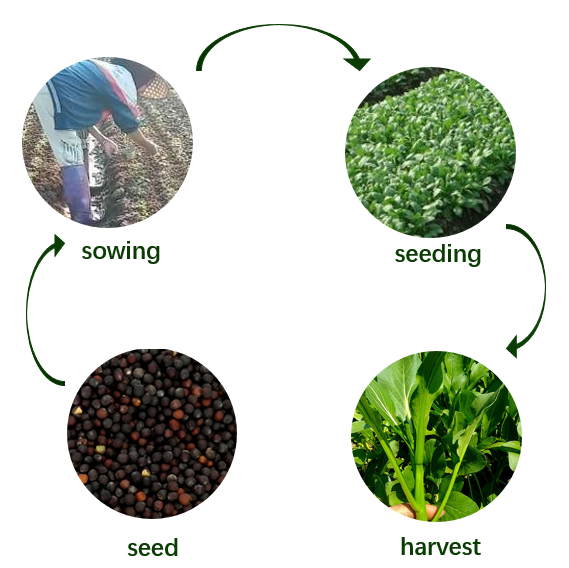


**Figure S4.** Chinese Flowering Cabbage cultivation growth period cycle. Chinese Flowering Cabbage is a leafy vegetable crop that edibles vegetable sprouts, and the cultivation is completed after vegetable sprouts harvest. Chinese Flowering Cabbage cultivation does not need to go through flowering and subsequent growth stages, and the growth period is short. In Ningxia, it can realize the cultivation of three cropping cycles a year, and it has become a common way of Chinese Flowering Cabbage cultivation.
